# The role of neurotrophic factors in retinal ganglion cell resiliency

**DOI:** 10.3389/fncel.2025.1536452

**Published:** 2025-01-29

**Authors:** Alan K. Abraham, Michael Telias

**Affiliations:** ^1^Department of Pharmacology and Physiology, University of Rochester Medical Center, Rochester, NY, United States; ^2^Flaum Eye Institute, University of Rochester Medical Center, Rochester, NY, United States; ^3^Center for Visual Science, University of Rochester Medical Center, Rochester, NY, United States

**Keywords:** retinal ganglion cell, neurotrophin, glaucoma, retinitis pigmentosa, brain derived neurotrophic factor, tropomyosin receptor kinase

## Abstract

Many retinal diseases are characterized by direct or indirect retinal ganglion cell (RGC) neurodegeneration. In glaucoma and optic nerve neuropathies, RGCs are the primary affected cells, whereas in photoreceptor dystrophies, RGC loss is secondary to the death of rods and cones. The death of RGCs in either case will irreversibly cause loss of vision, as RGCs are the sole output neurons of the retina. RGC neurodegeneration affects certain neurons preferentially, resulting in subpopulations of resilient and susceptible cells. Neurotrophins (NTs) are known to mediate neuronal survival through the downstream activation of various anti-apoptotic pathways. In this review, we summarize the current methods of RGC identification and quantification in animal models of direct or indirect neurodegeneration, and describe the advantages and disadvantages associated with these techniques. Using these techniques, multiple studies have uncovered the potential role of NTs in protecting RGCs during direct neurodegeneration, with BDNF and NGF delivery promoting RGC survival in models of experimental glaucoma. Many fewer studies have addressed similar questions in retinal diseases where RGC loss is secondary to photoreceptor degeneration, yielding conflicting results. Our analysis suggests that these seemingly contradictory results can be explained by the varying onset and geographic distribution of photoreceptor death.

## 1 Introduction

The retina is a multi-layered tissue containing various cell types that create circuits which detect light and transmit electrical signals. The various layers of the retina can be compartmentalized into the outer and inner retina. The outer retina contains rods and cones, the light-sensing photoreceptor cells, which connect to second-order neurons of the inner retina including horizontal, bipolar, amacrine, and Muller glial cells (Hoon et al., 2014; Malhotra et al., 2021; Kolb, 1995). These cells synapse onto the retinal ganglion cells (RGCs) at the inner plexiform layer (Todorova et al., 2022; Varshney et al., 2015). All signals initiated and transmitted by the phototransduction cascade eventually lead to RGC firing of action potentials, and the transmission by RGCs of visual information via the optic nerve (ON), to the brain (Rose et al., 2017; Mead and Tomarev, 2016). With RGCs being the only output neurons in the retina, RGC survival is necessary for vision preservation and restoration (Yuan et al., 2021).

During development, RGCs are first to differentiate from multipotent retinal progenitor cells through the activity of the transcription factor *Atoh7* (Lyu and Mu, 2021). The expression of various genes affects the physical properties and functions of RGCs, generating several different RGC subtypes, classified based on morphological features such as soma size and dendritic arborization, physiological responses to stimuli, and gene expression profiles (Rheaume et al., 2018; Laboissonniere et al., 2019; Tapia et al., 2022). The subtypes of RGCs differ among species, with approximately 20 and 40 subtypes identified so far in primates and rodents, respectively, (Kim et al., 2021; Langer et al., 2018; Jacoby and Schwartz, 2018). Following the processing of incoming visual signals, RGCs are responsible for relaying information to various locations in the brain. In mammals, the superior colliculus (SC) and lateral geniculate nucleus (LGN) are the primary recipients of RGC projections, but many other regions also receive input (Martersteck et al., 2017; Linden and Perry, 1983; Morin and Studholme, 2014). RGC subtype in the retina determine their axonal projections and influence the types of information relayed to the brain, including that related to attention, circadian rhythms, oculomotor control, and more (Goetz et al., 2022).

The direct or indirect loss of RGCs irreversibly leads to progressive vision loss. In diseases causing direct loss of RGCs, such as glaucoma, RGCs are the primary cells undergoing apoptosis, whereas in diseases causing photoreceptor loss, RGC neurodegeneration is secondary and downstream to the degeneration of other retinal neurons. Recent studies indicate that, regardless of how RGC loss occurs, some RGC subtypes are more resilient to neurodegeneration than others, suggesting the activation of a subtype-specific survival mechanism (Zhang et al., 2022b; Tapia et al., 2022; Duan et al., 2015). Here we review neurotrophins (NTs) and their potential role in mediating anti-apoptotic signals, promoting RGC survival and conferring a resilient cellular identity to a subset of RGCs.

## 2 Direct RGC neurodegeneration

RGC neurodegeneration is part of the natural aging process, with an approximate loss of 0.6% of RGCs per year in healthy humans (Harwerth et al., 2008). However, various animal models show no RGC loss with age, such as rats, which have stable RGC counts from a balance of retinal growth and density reduction, and marmosets (Harman et al., 2003; Nadal-Nicolás et al., 2018; Haverkamp et al., 2022). Therefore, findings in human retinas should be interpreted cautiously as quantification often relies on sampling and lacks the use of RGC-specific markers. Direct RGC degeneration is observed in glaucoma or ON injury, secondary neuritis such as in multiple sclerosis and other retinal and neurological diseases (Vernazza et al., 2021; Garcia-Martin et al., 2017). Of these, glaucoma is the most prominent disease, affecting 80 million people worldwide and is characterized by ON damage and the progressive death of RGCs (Tribble et al., 2023). Apart from childhood glaucoma, it is mostly an idiopathic disease, but the presence of single-nucleotide polymorphisms has been associated with increased risk (Kumar et al., 2024; Wang et al., 2022). Glaucoma is primarily associated with increased intraocular pressure (IOP) and/or reduced blood supply, activating apoptotic signals in RGCs (Qu et al., 2010; Zeitz et al., 2006). Although many treatments aim to reduce IOP, this strategy only hinders disease progression and does not reverse the RGC loss that prompted the initial diagnosis. Moreover, normal tension glaucoma presents RGC loss without an elevation in IOP, illustrating that IOP is not necessary for glaucoma pathogenesis (Anderson et al., 2001).

Glaucoma is studied in various animal models including non-human primates (NHPs), pigs, rabbits, and rodents (Evangelho et al., 2019). Although the NHP model provides the greatest anatomical similarity with the human eye, NHPs are often impractical for studies due to incomplete genomic data, long generation time, small litter size, high costs, and various ethical concerns. Pigs, rabbits, and rodents resolve the limitation of cost, but rodents prove to be the most feasible model due to fast generation time, general similarity with the human eye, and availability of diverse transgenic strains. Moreover, rodents such as the ground squirrel and tree shrew showcase visual streaks which resemble maculae, with tree shrews additionally possessing a lamina cribrosa, providing further anatomical similarities to the human eye (Xiao et al., 2021; Grannonico et al., 2024; Samuels et al., 2018). Despite their various advantages, rodent limitations include smaller overall eye size, making targets harder to access and manipulate, and the lack of a macula and lamina cribrosa in select rodents such as rats and mice (Chen and Zhang, 2015). Pressure-dependent rodent models of experimental glaucoma exhibit elevated IOP, caused by mutations or physical induction. For example, in the ocular hypertension model, magnetic microbeads are injected into the anterior chamber, leading to a sustained elevation in IOP for 4–6 weeks (Cwerman-Thibault et al., 2017). Other methods of IOP induction are intracameral injections, sclerosis of outflow pathway, cautery of extraocular veins, and transduction of trabecular meshwork with glaucoma associated genes (Pang and Clark, 2020). However, the more commonly used models have mutations resulting in elevated IOP, such as the DBA/2 mouse, carrying an inherited mutation in melanosomal protein genes *Tyrp1b* and *Gpnmb^R150X^* causing iris cell apoptosis (Cwerman-Thibault et al., 2017). This model develops elevated IOP between 6 and 9 months of age, resulting in RGC loss and reduced light responses (Amato et al., 2023; Inman et al., 2006).

A critical problem in characterizing glaucoma is quantifying RGC density. RGC quantification methods include immunostaining with specific antibodies such as RBPMS, Brn3a or Thy-1, and retrograde tracing from SC, LGN and other areas. Immunostaining presents a robust labeling technique, with RBPMS staining the majority of RGC subtypes in mice and Brn3a labeling 80% of RBPMS immunoreactive RGCs (Rodriguez et al., 2014; Kwong et al., 2010; Nadal-Nicolás et al., 2009). Thy-1 is less commonly used due to its lack of specificity, with additional labeling of amacrine cells, and reduction in expression before and during RGC loss (Lin et al., 2024; Dabin and Barnstable, 1995; Huang et al., 2006). Although RBPMS quantification is prone to counting error due to staining of overlapping cells with undefined borders, recent advances in artificial intelligence have improved quantification (Meng et al., 2024; Masin et al., 2021; Zhang et al., 2022a). Moreover, combining automated counting methods with other RGC specific antibodies such as Brn3a and POU6f2 will allow for more accurate total RGC counts (Lin et al., 2024). Altogether, immunolabeling provides reliable, consistent results in animal models but remains unsuitable for evaluating RGC survival in patients. Retrograde neuroanatomical tracing of RGCs, from the SC or LGN, using dyes such as cholera toxin subunit B and Fluorogold, or viruses, allows for reliable quantification of RGCs and enables highly detailed morphological assessment, but will fail to label RGCs projecting to other brain areas and requires invasive non-terminal surgical procedures in the mouse brain (Masin et al., 2021; Yao et al., 2018). Recent studies have utilized Fluorogold tracing from the intact ON and optic tract, providing the most accurate identification of the entire RGC population (Nadal-Nicolás et al., 2015). Moreover, RGC markers often have species-specific considerations, with certain markers being more effective in targeting RGCs within specific animal models (Nadal-Nicolás et al., 2023).

Quantification of RGCs via RBPMS immunofluorescence in the DBA/2 mouse model showed initial maintenance of RGC density in the central and peripheral retina at 2 and 6 months, but revealed a reduction of about 80% at 15 months (Amato et al., 2023). Another study in the same model compared RGC density with age-matched healthy mice, in which RGC loss began at 12 months of age and resulted in a 71% reduction in the DBA/2 mouse and 46% reduction in the healthy control after 18 months (Danias et al., 2003). RGC loss in the ocular hypertension model yielded similar outcomes with an expedited timeline, showing a 77% decrease in RGC density at 21 days post-injection, as measured by Brn3a immunolabeling (Trost et al., 2015). In both induced and genetic models of experimental glaucoma, late-stage degeneration results in significant RGC loss, with a reduction of approximately 70–80%. Moreover, RGC density is typically stable in early to mid-disease stages, with exponential reductions in RGC density observed in later stages, associated with the sustained elevation of IOP (Buckingham et al., 2008). Studies in rodent models of optic nerve crush have also found RGC subtype-specific resilience to degeneration, with intrinsically photosensitive RGCs demonstrating increased resilience and regeneration in response to damage whereas ON–OFF direction selective RGCs are more susceptible (VanderWall et al., 2020; Tapia et al., 2022). Together, this suggests the presence of a selective mechanism for degeneration in models of direct RGC neurodegeneration.

## 3 Indirect RGC neurodegeneration

**Figure 1:**
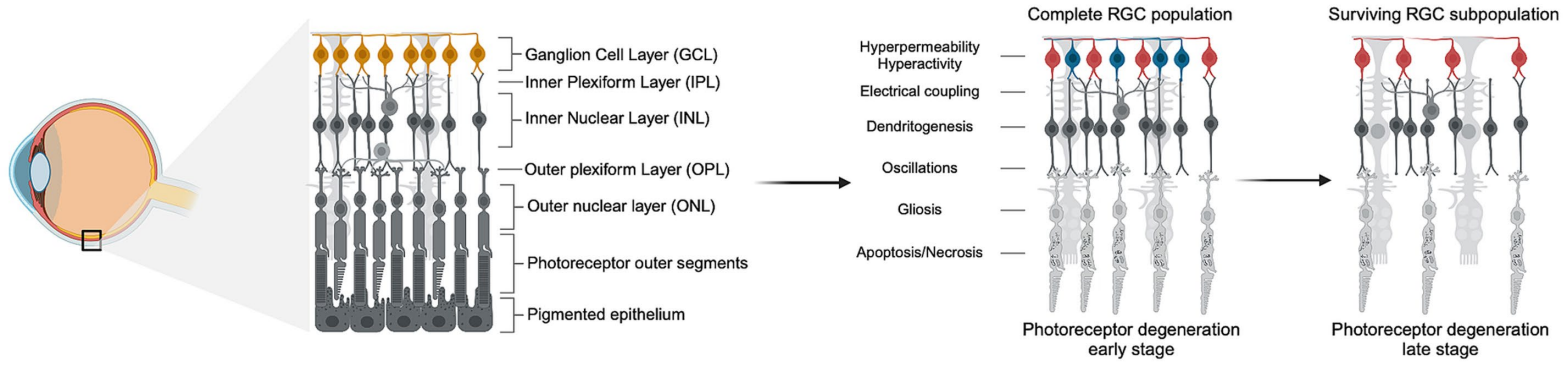
Photoreceptor degeneration leads to inner retinal remodeling and RGC loss. Schematic representation of the progression of photoreceptor degeneration characterized by the gradual loss of rods and cones. The left panel shows the anatomy of healthy retinal tissue with all the layers intact, and the position of the retina in the eye. The right panel depicts the progression of photoreceptor degeneration through early and late stages, showing the death of photoreceptors and the subsequent downstream pathophysiological remodeling in the layers of the inner retina, such as hyperpermeability and hyperactivity of RGCs. In early stages of photoreceptor loss, RGCs undergo remodeling but do not exhibit increased death rate. In late stages of photoreceptor loss and advanced remodeling, up to 80% of RGCs die but a subpopulation of resilient cells survive (depicted in red). *Created in BioRender. Abraham, A. (2024*), https://BioRender.com/l46y674.

Gradual RGC loss can be triggered by the primary death of photoreceptors. Photoreceptor loss can be idiopathic, such as age-related macular degeneration (AMD), or inherited, such as retinitis pigmentosa (RP). AMD affects nearly 200 million people worldwide and results from the gradual deterioration of the macula. Inherited retinal degenerations affect over 5.5 million people globally, with over 50 subtypes identified, of which RP is the most prevalent, affecting 1 in every 4,000 individuals (Ben-Yosef, 2022; Cross et al., 2022; Georgiou et al., 2021). In all forms of retinal degeneration, the sequential loss of photoreceptors leads to downstream pathophysiological changes in the inner retina known as remodeling, affecting RGCs, bipolar and amacrine cells (Figure 1) (Telias et al., 2020). One study showed that in advanced human cone-decimating RP, more than 80% of RGCs are lost, whereas in human cone-sparing RP in which central vision is preserved, RGCs seem to be maintained (Marc et al., 2003; Henriksen et al., 2014). Continued remodeling leads to the extensive loss of inner retinal neurons, including the loss of 70% of RGCs in RP, after which vision restoration is impossible (Pfeiffer and Jones, 2022; Santos et al., 1997).

Due to the complexities of RGC quantification previously discussed, studies in animal models of RP show conflicting results regarding RGC loss. One frequently used mouse model of aggressive RP is the RD1 model (*Pde6β^rd1/rd1^*), consisting of a mutation in phosphodiesterase 6β subunit found in rods, leading to initial photoreceptor loss by postnatal day (P) 21 and complete degeneration by P60 (Han et al., 2013). In this model, a longitudinal analysis of RGC density tracked using Thy-1 showed no significant changes in RGC density up to 18 months (Lin and Peng, 2013). However, as previously stated, Thy-1 may non-specifically label other inner retinal cells, suggesting that the stable RGC density observed might not be accurate. Another study, which utilized nuclear staining, concluded no significant differences in RGC density up to 12 months (Damiani et al., 2012). This conclusion is limited as RGCs were identified by nuclear morphology and similar RGC density was assumed across different retinal regions. Previous studies have established that density differs based on proximity and orientation relative to the ON (Dräger and Olsen, 1981). For example, RBPMS labeling in the RD1 model showed RGC density reduction of about 15% in the peripheral retina exclusively, as early as 3 months of age (Saha et al., 2016). However, it is possible that RBPMS preferentially labeled only a specific susceptible subpopulation of RGCs (Saha et al., 2016; Corral-Domenge et al., 2022; Stapley et al., 2022). By revealing axons, RGCs can be conclusively discriminated from other retinal cells, providing the most accurate identification method. Nevertheless, to date, no studies in RD1 mice or similar models have used axonal labeling to quantify RGCs.

Other commonly used models of RP are the P23H rat model, in which rhodopsin is misfolded, and the Royal College of Surgeons (RCS) rat model, in which the *Mertk* gene expressed in the retinal pigmented epithelium is mutated (Ryals et al., 2017; Orhan et al., 2015). The P23H model exhibits slow photoreceptor loss, with significant rod degeneration around P300, whereas the RCS model exhibits rapid photoreceptor loss as early as P18 (García-Ayuso et al., 2014; Sekirnjak et al., 2011). One study tracked RGC density in the P23H rat using Brn3a immunolabeling and Fluorogold retrograde tracing from the SC, showing an RGC density reduction of 15–20% in each label at 12 months (García-Ayuso et al., 2010). Another study in the same model found increased RGC degeneration through Brn3a labelling in the central retina as compared to the periphery, consistent with the pattern of photoreceptor loss (Kolomiets et al., 2010). Similar quantification of RGC density in the RCS model showed a 40% reduction after 18 months, with sections of the retina lacking both Fluorogold and Brn3a positive cells (García-Ayuso et al., 2014). Collectively, these studies suggest that the loss of RGCs may follow the geographic pattern of photoreceptor loss, indicating that a degeneration-dependent signal is affecting RGC survival.

## 4 Potential role of neurotrophins

NTs, such as brain derived neurotrophic factor (BDNF), nerve growth factor (NGF), neurotrophin-3 (NT-3), and neurotrophin-4/5 (NT-4/5), are growth factors which play essential roles in neuron development, proliferation, and survival (Colardo et al., 2021). NTs preferentially bind a class of transmembrane receptors known as tropomyosin receptor kinases (TRKs), with NGF binding TrkA, NT-4/5 and BDNF binding TrkB, and NT-3 binding TrkC (Lin et al., 2021). NTs are translated into premature peptides, or proneurotrophins, which are synthesized into their mature form by cleavage of the C-terminal domain. The canonical activation of TRKs results from mature NT binding, leading to the downstream activation of several pro-survival pathways, including phospholipase C-*γ* (PLC-γ), mitogen-activated protein kinase (MAPK), and phosphoinositide-3-kinase (PI3K-Akt) pathways (Uren and Turnley, 2014). Moreover, co-expression of nerve growth factor receptor (p75^NTR^) with TRKs can lead to high affinity binding of NTs, promoting synergistic downstream activation (Conroy and Coulson, 2022).

**Figure 2:**
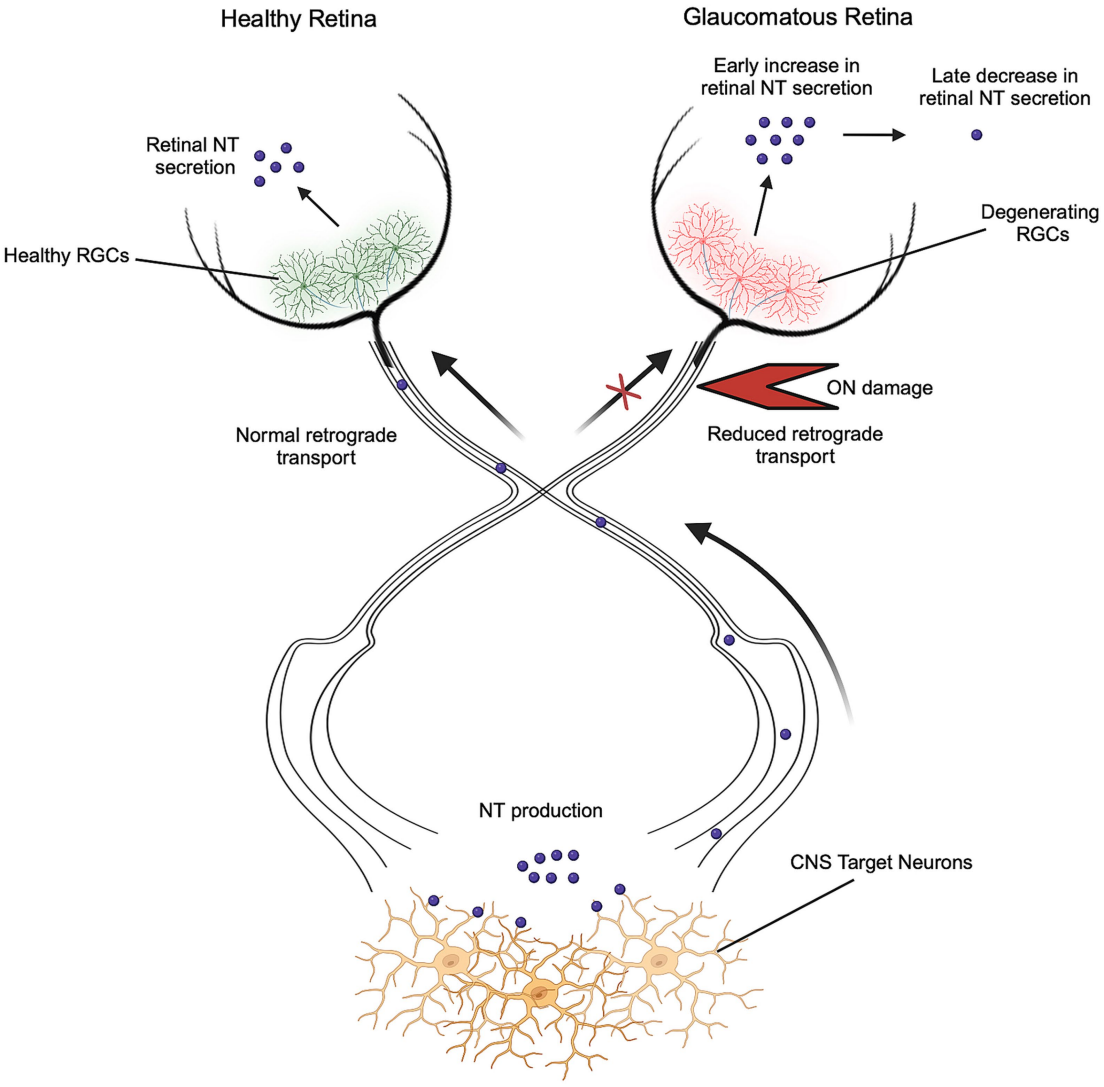
Neurotrophin deprivation in optic neuropathies. RGC survival is dependent on the continued activity of NTs, which are secreted locally in the retina and retrogradely transported from the brain. During glaucoma and other optic neuropathies, retrograde transport of NTs is disrupted due to ON damage, resulting in a reduced neuroprotective state. Reduction of retrograde sources of NTs triggers initial increases and later decreases in retinal NT secretion. *Created in BioRender. Abraham, A. (2024)*, https://BioRender.com/d88b197.

Alterations in NT secretion and activity characterize various neurodegenerative diseases such as Alzheimer’s, Parkinson’s, and Huntington’s disease, leading to a decline in long term potentiation, synapse formation, and neuronal survival (Bathina and Das, 2015). The effects of NTs in direct RGC neurodegeneration, such as glaucoma, are postulated by the NT deprivation hypothesis, which states that elevated IOP prevents the retrograde transport of NTs to RGCs, resulting in a reduced neuroprotective state (Figure 2) (Chitranshi et al., 2018). This hypothesis stems from a reduction in intraocular NT secretion during elevated IOP due to a block in NT transport at the ON head (Quigley et al., 2000). Deprivation of NT supply *in vitro* has been shown to induce up to 83% RGC loss observed after 48 h, suggesting that RGC survival is dependent on the activity of NTs (Johnson et al., 2024). Although the retrograde supply of NTs may be disrupted in glaucoma, many retinal cells have the capability of producing NTs. Murphy et al. showed that axonal lesions on RGCs targeting the SC at P5 caused significant RGC loss after 5 months, while lesions at P30 caused no loss, suggesting that adult RGC survival relies primarily on intraocular NT sources (Murphy and Clarke, 2006). Intraocular NT secretion has been observed in various studies, with BDNF expression occurring locally in astrocytes and RGCs (Gupta et al., 2014). Moreover, BDNF expression is locally upregulated in the ganglion cell layer immediately after ON injury (Pietrucha-Dutczak et al., 2018; Gao et al., 1997). The role of intraocular BDNF in neuroprotection was shown by inducing ocular hypertension in a mouse either homozygous or heterozygous for BDNF. The BDNF^+/−^ mouse showed a 40–45% reduction in GCL density and further reduced visual response as compared to the BDNF^+/+^ mouse, which showed a reduction of 30–35% (Gupta et al., 2014).

Since BDNF and other NTs are essential to RGC development and protection, many studies investigated the efficacy of introducing exogenous NTs to promote RGC survival. Intravitreal injections of recombinant BDNF in the ON crush cat model show positive correlations between dosage of BDNF injected and RGC density, with 30 μg treatments at the time of ON injury showing 81% RGC survival 1 week after administration (Chen and Weber, 2001). Moreover, higher doses of BDNF were associated with survival of medium-sized RGCs, likely due to this subpopulation comprising a larger proportion of all RGCs in the cat retina. Similarly, topical administration of NGF drops in rat IOP models resulted in greater RGC density after 7 weeks due to inhibition of apoptotic pathways, quantified by Bcl-2/Bax ratio (Lambiase et al., 2009). Treatment of three advanced glaucoma patients with the same NGF drops for 3 months resulted in progressive improvement in inner retinal layer function, post retinal neuronal conduction, contrast sensitivity, and visual acuity, all of which was sustained up to 3 months after discontinuation of treatment. Recent studies have initiated the Phase I clinical testing of topical NGF drops for glaucoma, deeming its application safe and tolerable at high concentrations (Beykin et al., 2022).

The limitations of topical NT administration, such as slow diffusion and short half-life (Wang et al., 2014) can be overcome by gene delivery through adeno-associated viral (AAV) vectors, which provide sustained upregulation and secretion. Various studies utilizing AAV-BDNF gene therapy have found success in sustained maintenance of IOP and reduced RGC loss. One study, which utilized a laser-induced rat IOP model, developed an AAV-BDNF, which resulted in 20% less RGC axonal loss after 4 weeks of elevated IOP (Martin et al., 2003). The sustained impact of BDNF delivery can be improved by studying the availability of its receptor TRKB during the progression of the disease. One study in microbead trabecular occlusion model of glaucoma found four-fold overall TRKB upregulation alongside reduction in BDNF (Wójcik-Gryciuk et al., 2020). In this study, moderate overexpression of AAV2-BDNF resulted in long-term RGC neuroprotection by restoring normal levels of TRKB expression. Moreover, rat models of induced IOP have increased activation of SH2 domain-containing phosphatase-2, a cytoplasmic protein which downregulates TRKB (Gupta et al., 2012). These studies suggest the importance of maintaining optimal BDNF and TRKB levels for therapies aimed at reducing RGC loss (Osborne et al., 2018).

The role of NTs in indirect RGC neurodegeneration has not been thoroughly studied. Some studies in the RD1 model have observed downregulation of TRKB, TRKC, BDNF, and ciliary neurotrophic factor (CNTF) (Xiaobei Yin et al., 2020). Intravitreal delivery of AAV-CNTF in the RD1 model showed increased photoreceptor layer thickness after 18 days (Cayouette and Gravel, 1997). Simultaneous treatment with recombinant CNTF and BDNF in RD1 retinal explants which were harvested at P2 and cultured for 9 days resulted in a reduction of TUNEL-positive photoreceptors and upregulated the activity of downstream survival factors such as Akt, ERK and CREB (Azadi et al., 2007). Similarly, supplementation of CNTF for up to 24 months in humans with RP through an encapsulated cell implant resulted in a dose-dependent increase in overall retinal and outer nuclear layer thickness (Birch et al., 2013). However, there was no change in thickness of photoreceptor outer segments and pigmented epithelium, and no improvement in vision. As none of these studies showed positive or negative effects on RGC density, further research on NT-mediated RGC survival in photoreceptor degeneration can promote the development of therapeutics to prevent vision loss and support restoration technologies reliant on RGC output. Previous studies suggest that retinal remodeling, which occurs during photoreceptor degeneration, triggers RGC axonal damage through vascular remodeling induced axotomy (García-Ayuso et al., 2011; Villegas-Pérez et al., 1998; Nguyen et al., 2023).

## 5 Conclusion

The degeneration of RGCs leads to irreversible vision loss, and since there is no current method of RGC regeneration, understanding their death is essential for vision restoration. RGC degeneration manifests in various conditions, with some leading to direct RGC loss and others resulting in RGC death secondary to other retinal pathologies. There exists much conflicting evidence regarding RGC loss during photoreceptor degeneration due to the absence of a gold standard technique for identifying RGCs. Moreover, both glaucoma and inherited photoreceptor degeneration are studied through a wide variety of animal models, with various advantages and disadvantages, further contributing to the discrepancies between studies. NTs regulate the development and survival of RGCs, making them candidates for neuroprotection. Their neuroprotective effect in direct degeneration may suggest that their application can be beneficial in RGC preservation during indirect degeneration as well.
